# Plastic Surgery in the Multimodal Treatment Concept of Soft Tissue Sarcoma: Influence of Radiation, Chemotherapy, and Isolated Limb Perfusion on Plastic Surgery Techniques

**DOI:** 10.3389/fonc.2015.00268

**Published:** 2015-12-01

**Authors:** Nicolai Kapalschinski, Ole Goertz, Kamran Harati, Maximilian Kueckelhaus, Jonas Kolbenschlag, Marcus Lehnhardt, Tobias Hirsch

**Affiliations:** ^1^Department of Plastic and Reconstructive Surgery, Burn Centre, BG University Hospital Bergmannsheil, Ruhr University Bochum, Bochum, Germany

**Keywords:** plastic surgery, sarcoma, radiation, chemotherapy, isolated limb perfusion

## Abstract

Surgical intervention is the mainstay treatment for soft tissue sarcomas (STSs). The significance of adjuvant and neoadjuvant therapies, such as chemotherapy, radiation, and isolated limb perfusion, remains under controversial discussion. The goal of this review is to discuss the effects of the aforementioned treatment modalities and their timing of application in plastic surgery techniques. Furthermore, options of reconstruction in cases of complications caused by adjuvant and neoadjuvant therapies are discussed. When compared with adjuvant radiation, neoadjuvant treatment can reduce negative side effects such as fibrosis and edema because radioderma can be removed during the subsequent surgical procedure. Furthermore, there have not been any reports of negative effects of neoadjuvant radiation on microsurgical procedures. However, the dose of neoadjuvant radiation correlates with increased risks of impaired wound healing postoperatively. Thus, a patient-specific approach to decide whether radiation should be performed adjuvant or neoadjuvant is necessary. Preoperative irradiation should be considered in cases where functional structures are exposed after tumor resection, in order to ensure the best possible functionality. Adjuvant radiation should be considered in all other cases because of its known superior wound healing. As for chemotherapy, no negative influence of its use adjuvant or neoadjuvant to reconstructive procedures, such as local or free flaps, has been reported. Lastly, small sample size studies have not shown increased risks of microsurgical failure or wound complications after isolated limb perfusion. The findings of this review suggest that the chronological order of the discussed therapeutic approaches is not a decisive factor in the surgical outcome of reconstructive procedures for STS.

## Background

Arising from the mesenchymal connective tissues, the heterogeneous group of soft tissue sarcomas (STS) occurs subcutaneous or deep in the extremities in 60% of cases (1). Surgical resection is the mainstay treatment of STS, and margin status is the most important prognostic factor. Margin status is usually documented according to the classification defined by Enneking et al. as intralesional (biopsy), marginal (resection through the pseudocapsule and pretumoral reactive tissue), wide (resection including surrounding “normal” tissue outside the reactive zone, but within the involved anatomical compartment), or radical (compartment resection) (2). Historically, amputation or compartment resection is most often chosen in order to ensure complete tumor removal. Modern limb salvage techniques combined with neoadjuvant or adjuvant radiotherapy are now the standard treatment options for extremity STS. Sparing adjacent critical structures is safe and contributes to improved functional outcomes (3). Nevertheless, due to the heterogeneity of histological STS subtypes and of responses to chemotherapy, radiation, and isolated limb perfusion, the significance of adjuvant and neoadjuvant options in multimodal therapeutic approaches were a controversial topic of discussion in the past (1). In 1982, Rosenberg et al. showed that there was no difference in local tumor control and disease-free survival between amputation and limb-saving surgery followed by radiation (4). In addition, further studies showed similar results for marginal, wide or radical resection incorporated into pre- and postoperative radiation (5, 6).

Sarcoma resection should be performed in specialized cancer centers. Preoperative diagnostic measures, such as magnetic resonance imaging (MRI), are indispensable to define the expected surgical margins. Tumor resection frequently affects exposed functional structures, such as bones, joints, tendons, blood vessels, or nerves in addition to soft tissue defects, introducing the need for reconstructive procedures that include local or free flaps and motor replacement surgery. Therefore, plastic surgery methods have to be included in the multimodal approach, and the preference is shifted from conventional simple techniques of wound closure toward microsurgical procedures that enable the transfer of uninvolved tissues to the affected region. Furthermore, reconstructive surgery can help treat complications caused by adjuvant and neoadjuvant therapies. The timepoint at which additional treatments are introduced directly affects the planning for and the outcomes of tumor resection and reconstructive plastic surgery techniques.

The goal of this review is to show the influence of the timing of introduction of additional treatments for STS, such as radiation, chemotherapy, and isolated limb perfusion, on the surgical techniques used for reconstruction following tumor resection.

## Radiotherapy

Despite years of experience, the role of radiotherapy in the treatment of extremities STS was not fully established in the past (1). It has been demonstrated that neoadjuvant irradiation provides no significant benefit in local control of the tumors and/or development of distant metastasis when compared with adjuvant treatment (6, 7). Moreover, the influence of neoadjuvant irradiation on subsequent plastic surgery techniques remains controversially discussed. As radiation theoretically sterilizes the reactive zone surrounding the tumor, neoadjuvant radiation may allow marginal excision to be safely performed around vital structures without compromising local control rates (6). Neoadjuvant irradiation enables a smaller radiation field size when compared with adjuvant irradiation (8).

After neoadjuvant radiotherapy, complete removal of surrounding radioderma prior to soft tissue coverage via plastic surgery can be performed. As a result, late radiation effects, such as fibrosis, caused by increased collagen synthesis as a side effect of external radiotherapy and edema can be reduced. This procedure is of major interest in cases of exposed functional structures, such as tendons or joints where fibrosis and edema can compromise functional restrictions. Furthermore, preoperative treatment prevents the delay between irradiation and surgical resection that is caused by a possible compromised wound healing when radiotherapy is performed adjuvant. Several studies showed that neoadjuvant radiation has no negative effect on microsurgical procedures, including free flaps (9–11). For afferent vessels located in postradiogenic altered tissue, there was no significant increase of complications. Even though preoperative radiotherapy typically involves a lower dose of radiation when compared with postoperative radiotherapy, the risk of impaired postoperative wound healing rises in direct correlation with the neoadjuvant radiation dose (12). O’Sullivan reported that 35% of patients receiving preoperative radiotherapy (50 Gy in 25 fractions) and 17% of patients receiving postoperative radiotherapy (66 Gy in 33 fractions) developed wound complications irrespective of the surgical procedure (primary closure or plastic surgery) (7, 12). Other authors reported that 21–37% of the patients who received neoadjuvant radiation developed serious local wound complications including infection, tissue necrosis, seroma, and dehiscence, particularly at the proximal lower extremity (36 vs. 15–27% for other locations) (6, 13). Interestingly, for the upper extremity, much less wound healing problems were described compared to the lower extremity after neoadjuvant radiation therapy (7). Thus, a different regimen of therapy chronology can be considered for the upper extremity. Nevertheless, additional surgical procedures aimed to control wound morbidity were necessary in 16–23% of the cases (13, 14). On the other hand, rates of fibrosis were increased in patients who received adjuvant treatment. Radiation doses of 50–60 Gy within 6 weeks after surgery can reduce local recurrence, but fibrosis and postradiogenic altered skin can provoke chronic ulceration in an otherwise adequately healed transplant (15). Resulting unstable scars and fibrotic tissue may cause inferior functional outcomes (16).

High-dose-rate brachytherapy provides a constant dose to the target and very low doses to nearby radiosensitive tissues. Sharma et al. reported that perioperative high-dose-rate interstitial brachytherapy in combination with external beam radiation therapy provides excellent local control and survival rates (follow-up 46 months) with acceptable acute and late toxicities (17). Delayed wound healing was observed in 5.7% of cases, whereas chronic skin lesions and fibrosis were observed in 9.6% (17). Nevertheless, other studies demonstrated an increased rate of impaired wound healing after brachytherapy combined with neoadjuvant radiation when compared with external radiation, irrespective of the reconstructive approach (18). The overall higher radiation dose might be an explanation for these findings. No negative influence to microsurgical reconstruction has been described (19). However, it is important to note that the source of radiation should be placed at the maximal distance possible to the anastomosis because local radiation treatment decreases vessel wall strength (20).

## Chemotherapy

Just as in the case of radiotherapy discussed above, the role of adjuvant or neoadjuvant chemotherapy in the treatment of STS is controversial due to the low response rate and toxic adverse effects. Marginal positive effect to the disease-free surviving was reported in several meta-analyses in the past (21). Nevertheless, for first line therapy including Doxorubicin and Ifosfamide, a response rate of up to 30% is described while no benefit in relapse-free survival or overall survival could be shown (22, 23). Based on these findings, adjuvant chemotherapy should be limited to high-risk patients with extensive, highly malignant STS in the setting of controlled clinical studies (23).

There have been no reports to the authors’ knowledge of negative influence of chemotherapy on reconstructive procedures involving local or free flaps. However, it is noted that adjuvant treatment should not start before completion of wound healing because there is a known increased risk of wound complications associated with chemotherapy (6). Sanniec et al. showed wound complications in 49% of cases of combined sarcoma resection and chemotherapy (24).

Extravasation of cytostatic drugs during peripheral intravenous administration is a potentially severe complication of chemotherapy. The frequency of extravasation is considered to be between 0.6 and 6% (25). The extent of local tissue injury depends on the chemical structure of the applied substance, which is classified as vesicant, irritant, and non-irritant. According to current references, treatment of chemotherapy drug extravasation should be managed in specialized centers (26). Surgical interventions are indicated in cases of extensive necrotic areas or failure of conservative measures. After surgical debridement, reconstructive procedures, such as split-thickness skin grafts or randomized fasciocutaneous flaps, prevent prolonged secondary wound healing. Thus, plastic surgical treatment enables early and complete remission of the lesions and reduces the delay in administration of further chemotherapy (27).

## Isolated Limb Perfusion

For the treatment of locally advanced STS in the extremities, isolated limb perfusion with TNF-alpha and melphalan (TM-ILP) has proven to be a effective treatment modality with limb salvage rates of ~87% and response rates of 71% (28, 29). Tumor size reduction induced by TM-ILP can render non-resectable tumors to resectable.

Unfortunately, there is limited available data for reconstructive procedures following isolated limb perfusion. Even when local complications, such as impaired wound healing and lymphocutaneous fistulas are described, small sample size studies demonstrated no increased risk of microsurgical failure or wound complications (30). Functional results after limb-sparing surgery were shown to be satisfactory (31).

## Conclusion

**Figure 1:**
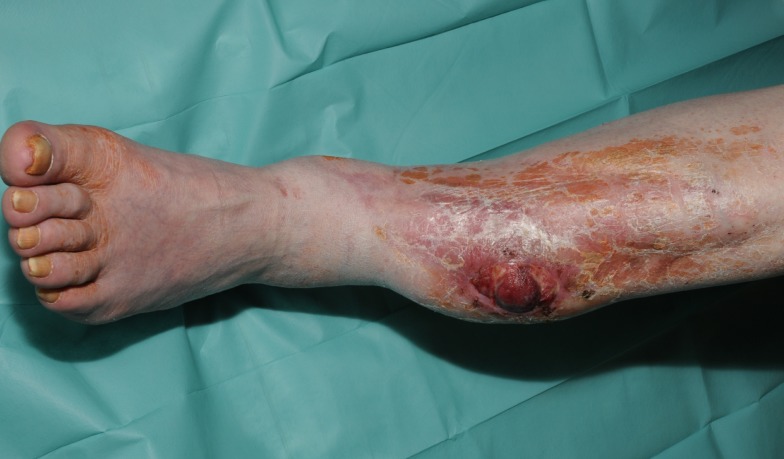
**Recurrence of a myofibroblastic sarcoma (TNM classification: pT2b pNX M0, G1) of the left lower leg 13 years after tumor resection and free latissimus dorsi transfer**. Status post neoadjuvant isolated limb perfusion.

**Figure 2:**
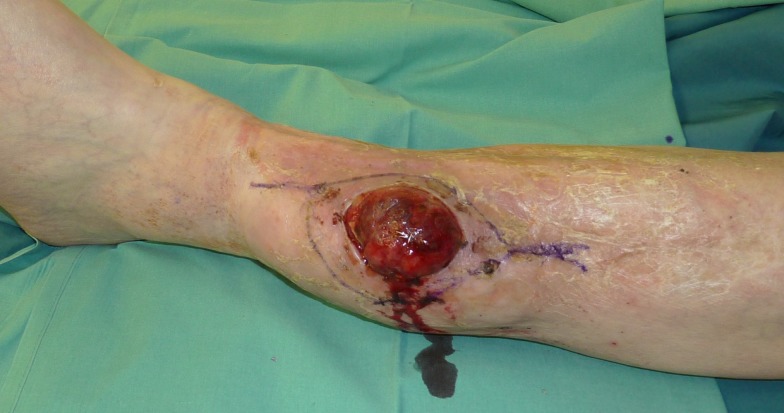
**Preoperative planning of tumor resection (R0)**.

**Figure 3:**
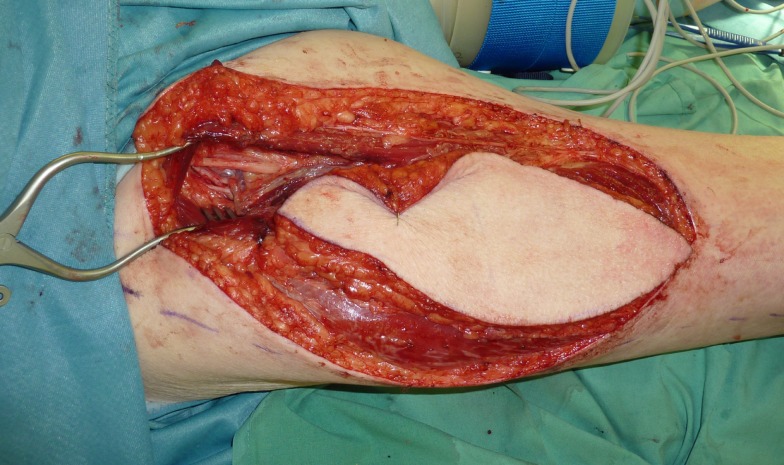
**Elevation of an anteriomedial thight (AMT) flap and dissection of the pedicle**.

**Figure 4:**
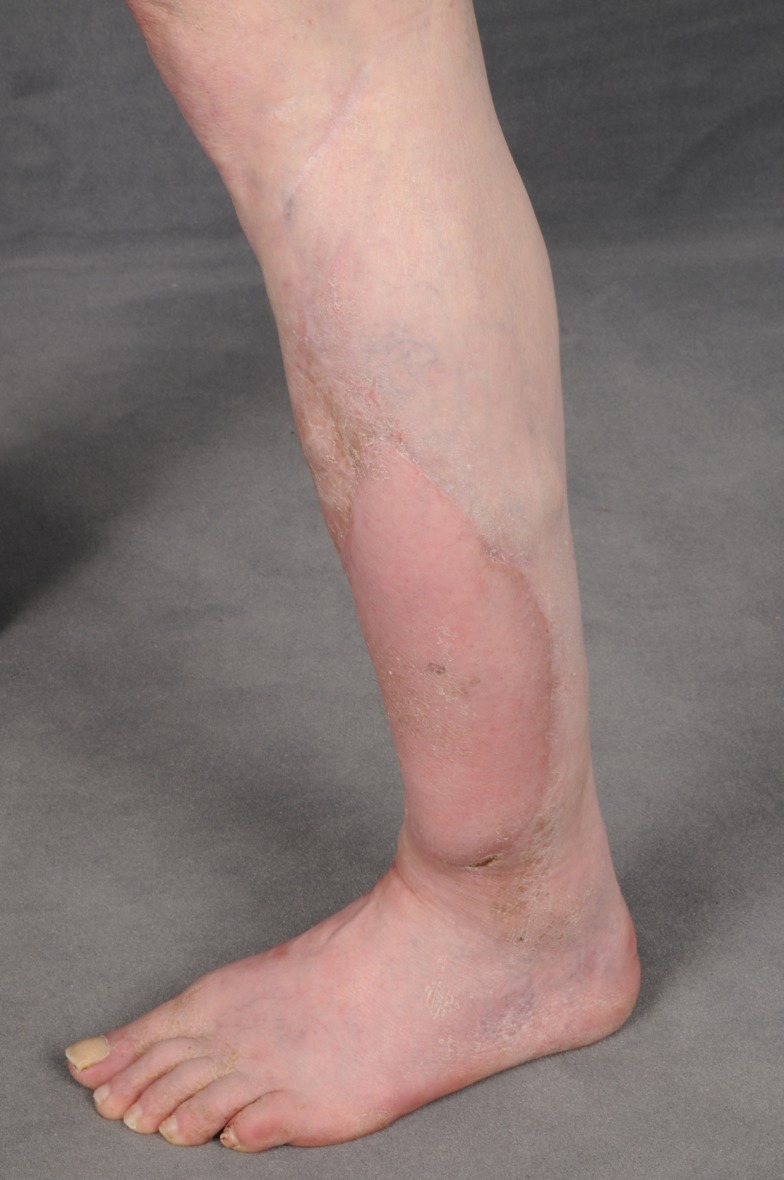
**Postoperative result after complete wound healing**.

Interdisciplinary treatment involving pathologists, radiologists, surgeons, radiation therapists, and medical oncologists is mandatory in cases of STS. Therapy planning and performance should be carried out at referral centers for sarcomas that can provide the necessary multidisciplinary environment. Irradiation represents a mainstay in treatment. The significance of chemotherapy and isolated limb perfusion on the outcomes of reconstructive procedures following STS resection is yet to be elucidated. The chronology of radiation, chemotherapy, limb perfusion, and surgical resection appears to have no influence on the success of reconstructive procedures, such as microsurgical tissue transfers (Figures 1–4). Most surgeons prefer postoperative radiation because it may afford decreased risks of wound complications. In cases of neoadjuvant radiation, free flaps may be performed for soft-tissue reconstruction. Several studies showed superior functional outcomes with preoperative, rather than postoperative radiotherapy. Pre- and postoperative irradiation mainly affects plastic surgery procedures in terms of impaired wound healing and fibrosis. Further studies will be necessary to elucidate the effects of chemotherapy and isolated limb perfusion on the outcomes of reconstructive procedures following resection of STSs.

## Conflict of Interest Statement

The authors declare that the research was conducted in the absence of any commercial or financial relationships that could be construed as a potential conflict of interest.
